# Information Seeking and Engagement on Social Media Among Pediatric Solid Organ Transplant Recipients and Their Families

**DOI:** 10.1111/petr.70480

**Published:** 2026-10-08

**Authors:** Irene Chen, Christopher Yu, Julie M. Robillard, Tom Blydt‐Hansen

**Affiliations:** ^1^ Department of Pediatrics University of British Columbia Vancouver British Columbia Canada; ^2^ BC Children's & Women's Hospitals Vancouver British Columbia Canada; ^3^ Division of Neurology University of British Columbia Vancouver British Columbia Canada

## Abstract

**Background:**

Social media is widely used among adolescents and offers unique opportunities to support children with chronic illnesses. However, little is known about how pediatric solid organ transplant (SOT) recipients and their families engage with these platforms. The objectives of this study are to characterize how this population seeks and shares information and engages with other pediatric SOT recipients and their families or general members on a transplant‐specific online group.

**Methods:**

We conducted a comprehensive search of the Reddit group, r/transplant. Publicly available posts and comments were included if they contained a self‐identification as a pediatric SOT recipient (0–24 years old) or a family member. An inductive content analysis was performed to identify key topics. Self‐disclosed demographic information was used to explore differences in discussion topics and engagement.

**Results:**

A total of 357 posts and 5827 comments met inclusion criteria. Users most commonly discussed medical symptoms, medication, social support, healthcare experiences, and post‐transplant recovery. Family members predominantly focused on medical issues, while recipients addressed a broader range of topics. Topics users engaged with varied by time since transplant: recipients 0–4 years post‐transplant frequently discussed emotional and medical challenges, while those 5+ years post‐transplant posted more about celebrating milestones.

**Conclusions:**

Pediatric SOT recipients and their families use social media to seek and share information. Engagement patterns differ between recipients and their family members and across years post‐transplant, suggesting the potential for tailored online interventions to support evolving needs across the transplant journey.

## Graphical abstract

Pediatric SOT recipients and families use r/transplant to share information on medical, mental health, and psychosocial topics. Differences in post content by user role and time since transplant highlight the need for tailored online support resources.

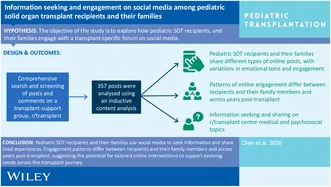

**Abbreviations**
CKD: chronic kidney disease
HCP: healthcare practitioner
QoL: quality of life
SOT: solid organ transplantation

## 1 Introduction

Solid organ transplantation (SOT) is a life‐saving procedure for children with end‐stage organ failure [1]. As survival rates have increased, there has been a growing emphasis on enhancing Quality of Life (QoL) and psychosocial well‐being of SOT patients. While QoL generally improves after a transplant, it remains impaired in several key areas, such as physical health, psychosocial health, social interactions, and school performance, when compared to peers without a SOT [2, 3].

Notably, despite experiencing a greater sense of normalcy and improved medical outcomes, SOT patients report continued discomfort and disappointment due to immunosuppression side effects, as well as feelings of inferiority related to physical limitations [4]. In fact, the QoL of children living with a transplant can be comparable to that of other pediatric chronic illness populations, including children with cancer in remission [5].

Qualitative research highlights a strong desire for normalcy in adolescents and youth [6, 7]. Physical changes resulting from immunosuppressants or surgeries, daily medication adherence, regular clinic visits and medical tests, extended absences from school, and disruptions in peer relationships: these are all aspects of the pediatric transplant experience that hinder this pursuit of normalcy, potentially fostering feelings of inadequacy and contributing to poor mental health [1, 8, 9]. Despite a growing understanding of the psychosocial challenges faced by pediatric SOT recipients, there are significant gaps in targeted, evidence‐based resources for this community [1].

The use of internet and social media has become ubiquitous in the lives of young people, with over 95% of adolescents having access to a smartphone and 45% reporting that they're online “almost constantly” [10]. In adolescents with chronic diseases, social media platforms offer valuable spaces to build social connections, share experiences, and gather disease‐specific knowledge [11, 12] For instance, De Nardi and colleagues found that over 94% of adolescents with chronic conditions turned to Facebook to connect with peers facing similar challenges and frequently searched the internet for disease‐specific information [13]. The majority of parents and caregivers of children with cancer also report using social media to provide and receive social or emotional support [14].

In pediatric SOT recipients, digital tools and mobile applications have shown promise in encouraging medication adherence [15]. Virtual peer support spaces for young people living with a SOT have also demonstrated high engagement and acceptability, with the potential to enhance a sense of normalcy, self‐identity, community contribution, and social connectedness [16, 17]. This holds significant potential for pediatric SOT recipients, as feelings of normalcy and social support are determinants in QoL, medication adherence, and mental health outcomes.

Existing literature highlights the promising role of social media in supporting young transplant recipients. One case study describes a pre‐heart transplant patient who used social media to reduce feelings of isolation and develop health literacy [18]. Caregivers of pediatric transplant recipients also report high social media use, with over 40% stating they received COVID‐19‐related information through these platforms [19]. Similarly, another study found that 69% of young patients with chronic kidney disease (CKD) and their parents recognized social media as an important source of support and disease management [20]. In other adolescent and young adult chronic disease populations, social media‐based platforms and tools have been shown to enhance self‐management, supplement disease‐specific knowledge, and empower users through community support [21, 22]. Despite the potential for social media‐based tools to support psychosocial well‐being, our understanding of how pediatric SOT recipients and their families engage with social media remains limited.

The use of social media posts and comments as units of analysis offers unique insights into the lived experiences of pediatric SOT recipients, including children, adolescents, young adults, and their families, that may not be captured by traditional data collection methods. For example, the analysis of social media posts and hashtags has been employed to examine health issues, such as attitudes towards vaccine uptake, organ donation, child pain, and experiences with anxiety, trauma, and disordered eating [23, 24, 25, 26, 27, 28]. Reddit, a popular online platform, is particularly relevant in this context. It allows users to engage in discussions, share content, and participate in subreddits dedicated to specific topics [29, 30]. Users can create pseudonymous usernames, ensuring anonymity and fostering open discussions. The transplant‐specific subreddit, r/transplant, which has approximately 13 000 members, stands out as one of the largest support communities for transplant recipients on social media.

The objective of the current study is to explore how pediatric SOT recipients and their families engage with a transplant‐specific forum on social media. Specifically, we sought to characterize major categories of information exchanged and engagement with these topics in this community. Moreover, this study aims to provide healthcare providers (HCPs) with a deeper understanding of the peer‐to‐peer support and informational landscape their patients and families may be engaging with, which may inform clinical conversations and resource recommendations.

## 2 Methods

### 2.1 Study Design

This study employed a qualitative content analysis of social media posts and comments. Data were sourced from public posts made by self‐identified pediatric transplant recipients and their families on r/transplant on Reddit. As a part of the content analysis procedure, a coding guide was developed to capture the major topics present in the content. Posts were organized into major categories to assess for differences in engagement and sentiment associated with various topics. Self‐disclosed demographic information was used to explore differences in discussion topics and levels of engagement.

#### 2.1.1 Ethical Considerations

Based on the UBC Research Ethics Board guidelines for research using social networking sites and Article 2.2 of the Tri Council Policy Statement, which states that “research does not require REB review when it relies exclusively on information that is in the public domain and the individuals to whom the information refers have no reasonable expectation of privacy,” ethics approval was not sought for this study. r/transplant is a publicly accessible forum where anyone can view posts without needing to register, log in, or request membership. When users post on the subreddit, they do so with the understanding that their content is visible to the general public. This study involved no intervention or direct contact with users and relied purely on observation of existing, public content. Therefore, based on UBC REB guidelines and TCPS 2 Article 2.2(b), formal REB review was not required.

To further protect against re‐identification, any demographic variables collected with an n‐value of less than 5 were removed. All quotes presented were reviewed to ensure they don't contain identifying information and any personal details shared (i.e., age at transplant) were replaced with the broader category. We also did not use any deleted posts or comments. We further acknowledge that we did not engage with the forum's moderators or users to gather feedback on our coding or interpretations. This was a deliberate choice to honor the forum as a patient‐led space and avoid any potential for undue influence or intrusion.

This research was conducted in accordance with Reddit's User Agreement and Terms of Service, which permit the analysis of publicly available content. Our data collection and analysis methods were designed to respect the autonomy of the community and the individuals who contribute to it.

#### 2.1.2 Reflexivity

The lead researcher is a female graduate student with no lived experience of having a pediatric SOT or a pediatric chronic illness. An undergraduate health sciences student contributed to the analysis, specifically as a second independent coder in the qualitative analyses. Investigators of the study include a Scientist in Patient Experience, with experience in research related to social media and health technology, and a Pediatric Nephrologist, with experience providing care to young people living with a transplant and their families.

### 2.2 Setting

#### 2.2.1 r/Transplant Group

Reddit is a popular online platform where users can create and participate in communities centered around specific topics of interest. These communities, known as “subreddits,” are organized by topic and allow members to engage in discussions. The subreddit, r/transplant, is the largest transplant‐specific subreddit on the platform, with approximately 13 000 members as of June 2025 and was created in April 2013.

### 2.3 Study Cohort

Posts shared in r/transplant (January 22, 2014–January 22, 2024) were comprehensively scraped using the RedditExtractoR package in R Studio and an adapted Reddit extraction script in Python. Post data (title, content, URL, timestamp, number of comments, and upvote count) was exported into Excel files. Only text was extracted and evaluated; visual content in posts was not analyzed.

The dataset was initially screened for deleted posts, posts shared by users with deleted accounts, posts removed by moderators, and duplicate posts. Posts were excluded if they were made by individuals who were not SOT recipients or immediate family members of an SOT recipient.

For inclusion in the analysis, posts had to be shared by users who self‐identified as SOT recipients or from users awaiting a transplant. Posts were excluded if they did not disclose that they received their transplant between the ages of 0–24 years. Posts from family members of transplant recipients meeting these criteria were also included. Eligible family relationships included parents, siblings, grandparents, aunts or uncles, cousins, children, and spouses or partners.

Comments under posts that met the inclusion criteria were included in the analysis to specifically assess information sharing and engagement between users. However, these comments were not included in the main analysis of topics and usage patterns, as we were unable to verify that commenters were pediatric recipients or their family members. For both family members and recipients, multiple posts or comments from the same user, identified by unique usernames, were included.

All posts were manually screened using the post content, title, and/or information available on the user's profile.

### 2.4 Demographic Variables and Engagement

Users are not required on the subreddit to disclose demographic information. When disclosed, the user's gender, type of organ transplant received, age at transplant, years post‐transplant, and current age were collected. For posts from family members, the type of familial relationship and any disclosed characteristics of the transplant recipient were recorded.

Data on user engagement were also collected, including the number of upvotes and the number of comments each post received. Both comments and upvotes indicate greater discussion surrounding post content and boost post visibility on the Reddit page. Upvotes are similar to “likes” on other social media platforms and can convey support or approval for the post or interest in its content.

### 2.5 Data Sources and Measurement

Posts and comments were analyzed in NVivo 14. As a part of the content analysis procedure, a coding guide was developed to systematically organize the content of posts and comments in r/transplant.

The coding guide was refined through an inductive and iterative process. First, a subset of 50 posts was independently read and open‐coded by two coders (IC and CY). These initial codes were discussed, compared, and grouped into preliminary themes. This preliminary guide was then applied to a new subset of 50 posts. The coding team met biweekly to discuss new or conflicting codes, refining the thematic structure. The Cohen's kappa coefficient was calculated after each round to assess the agreement in coding between independent coders. This iterative process of independent coding, review, and revision continued until a Cohen's kappa coefficient of greater than 0.75 was achieved. A total of 7 rounds of coding refinement were conducted, which included 67 posts that were coded by both reviewers. In cases where a consensus couldn't be reached, senior members of the research team were consulted. Once agreement was established, the entire dataset was split between the two coders. Posts were recoded using the final coding guide, with new emerging codes added and discussed. The final coding guide is provided in Table S4.

### 2.6 Quantitative Variables

#### 2.6.1 Analysis of Engagement and Sentiment

Differences in number of comments, number of upvotes, and sentiment score between major post categories and demographic categories were assessed using an independent samples *t*‐test or one‐way factorial ANOVA in Excel.

Sentiment score was derived using the Tidy Approach to Text Mining with R [31]. This approach uses the programming language R to tokenize each word within the text body that is then individually tagged with a value. The score for each post and comment was calculated by aggregating each token within the text. The dataset used to score each word selected for this study was the AFINN sentiment analysis initially developed by Finn Arup Nielsen, which contains 2476 words valued between −5 and +5 to represent negative and positive valence, respectively.

#### 2.6.2 Subgroup Statistical Analysis

Numerical values including, sentiment score, number of comments, and number of upvotes on posts, were analyzed using Microsoft Excel and IBM SPSS Statistics 29.0. Posts were grouped into 5 major categories, which were created based on the most frequently referenced personal experience codes identified from the content analysis. These include the following:

1. *Medical or medication*,
2. *Mental health or emotional struggles*,
3. *Interpersonal relationships*,
4. *Long‐term outcomes of transplant*, and
5. *Celebrating a transplant milestone*.

Posts were independently categorized by two coders, and only those with unanimous agreement were included in further analysis. Posts that did not fit into any of these categories were excluded. Each post was assigned to only one category. While codes informed the development of the 5 major categories, the designation of a post to a category was independent of the codes assigned to segments of text in a post or comment.

Differences in mean sentiment score, number of comments, and number of upvotes were assessed using a one‐way ANOVA. When significant (*p* < 0.05), a Tukey's HSD was performed as a post hoc analysis to identify which categories differed significantly.

Differences in the number of posts in each of these 5 categories between groups (based on gender, type of organ received, years' post‐transplant, and age at transplant) were assessed using the Likelihood Ratio Chi‐Squared test. When significant (*p* < 0.05), a *z*‐test for proportions was used to identify specific differences between groups. Group differences in mean number of comments and upvotes were assessed using either an independent samples *t*‐test (for 2 groups) or one‐way ANOVA (for 3+ groups) followed by a Tukey's HSD for post hoc comparisons.

## 3 Results

### 3.1 Sample Determination

A comprehensive search of r/transplant between January 22, 2014 and January 22, 2024, yielded 8393 posts (Figure 1). Of these posts, 1035 posts were deleted, posted by a deleted account, duplicates, or removed. Of the remaining 7358 posts, 7001 posts were excluded because the poster was not a SOT recipient, did not receive a transplant between the ages of 0 and 24 years, or did not disclose their age at transplant. The remaining 357 posts and 5827 comments left under these posts were included in the analysis.

**FIGURE 1:**
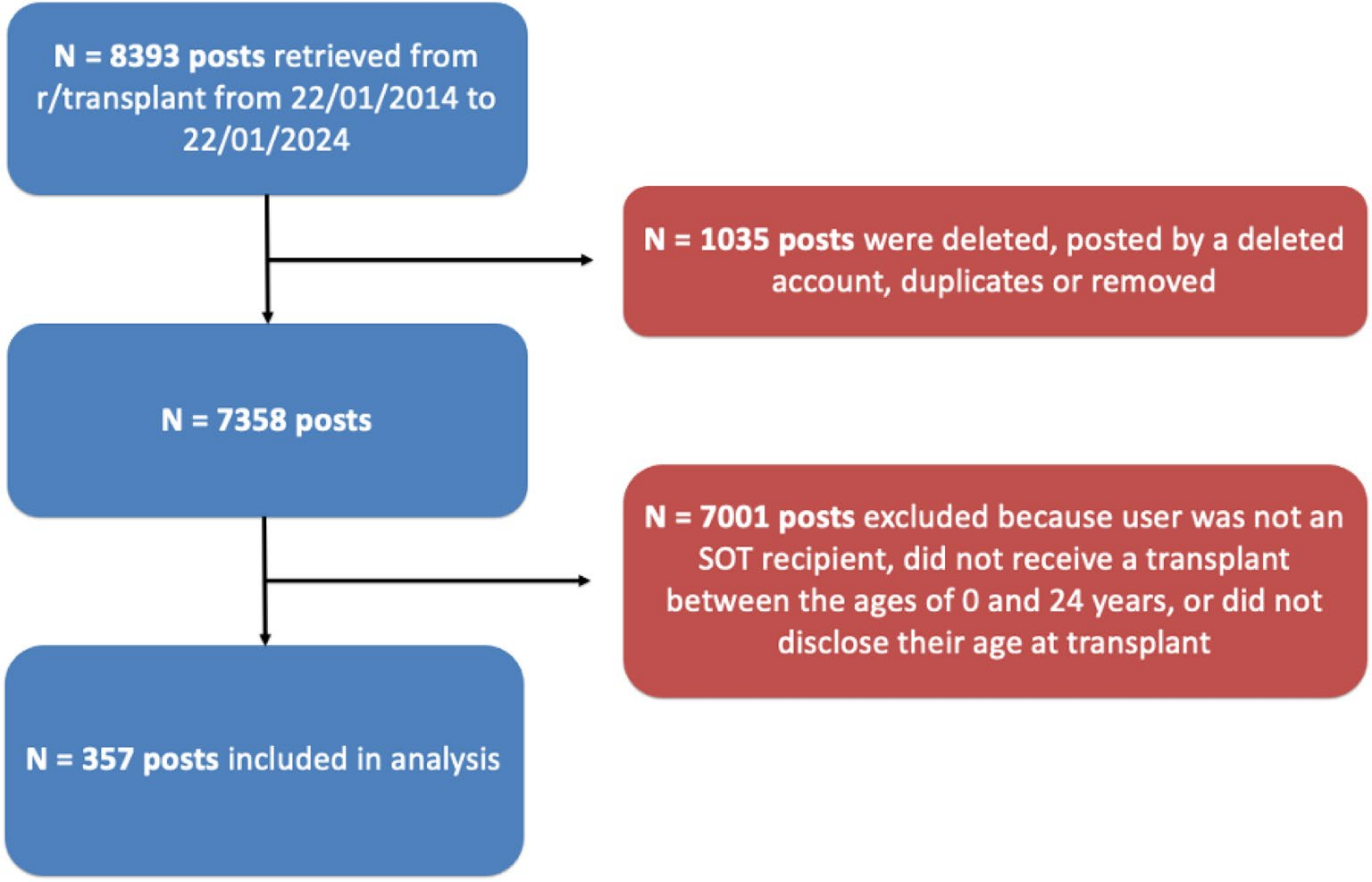
Screening of posts from r/transplant.

### 3.2 Descriptive Data

#### 3.2.1 Transplant Recipient Posts

User age at posting was disclosed in 65% of posts (*n* = 195), with the most common age at posting being 20–24 years (*n* = 97). Of those that disclosed gender, 19% of users (*n* = 58) identified as a man or boy (*n* = 30, 52%) or a woman or girl (*n* = 28, 48%), with no other gender identities self‐reported (Table 1).

**TABLE 1:** Demographic characteristics of transplant recipient posts that met inclusion criteria.

Posts from transplant recipients	
*n*	301
Age of user	
Disclosed	195 (65%)
13–19 years	23 (12%)
20–24 years	97 (50%)
25+ years	75 (38%)
Not disclosed	106 (35%)
Gender	
Disclosed	58 (19%)
Man or boy	30 (52%)
Woman or girl	28 (48%)
Not disclosed	243 (81%)
Organ	
Disclosed	206 (68%)
Kidney	86 (42%)
Heart	57 (28%)
Liver	42 (20%)
Lung	17 (8%)
Other	4 (2%)
Not disclosed	95 (32%)
Age at Tx	
Disclosed	124 (41%)
0–9 years	39 (31%)
10–19 years	67 (54%)
20–24 years	18 (15%)
Not disclosed	177 (59%)
Years after Tx	
Disclosed	148 (49%)
0–4 years	43 (29%)
5+ years	105 (71%)
Not disclosed	153 (51%)

Organ type was shared in 68% posts (*n* = 206), with the most common transplants being kidney (*n* = 86, 42%). Age at transplant was reported in 41% posts (*n* = 124), with users most frequently reporting that they received their transplant between the years of 10–19 (*n* = 67). Years post‐transplant was reported in 49% of posts (*n* = 148), with 29% (*n* = 43) in their first four years post‐transplant and 71% (*n* = 105) living with their transplant for five or more years.

#### 3.2.2 Family Member Posts

Among the 56 posts from family members, parents were the most represented group (*n* = 36, 64%), followed by siblings (*n* = 14, 25%) (Table 2). Gender of the family member with the transplant was reported in 35 posts (63%), with 54% identifying as a man or boy and 46% as a woman or girl. No other gender identities were disclosed.

**TABLE 2:** Demographic characteristics of family member posts that met inclusion criteria.

Posts from family members	
*n*	56
Family member relationship	
Parent	36 (64%)
Sibling	14 (25%)
Partner	5 (9%)
Other	1 (2%)
Gender	
Disclosed	35 (63%)
Man or boy	19 (54%)
Woman or girl	16 (46%)
Not disclosed	21 (38%)
Organ of family member with Tx	
Disclosed	37 (66%)
Liver	16 (46%)
Heart	13 (35%)
Other	8 (22%)
Not disclosed	19 (34%)
Age of family member with Tx	
Disclosed	34 (61%)
0–9 years	13 (38%)
10–19 years	13 (38%)
20+ years	8 (24%)
Not disclosed	22 (39%)
Years after Tx	
Disclosed	17 (30%)
0–4 years	8 (47%)
5+ years	9 (53%)
Not disclosed	39 (70%)

The organ received by the family member was disclosed in 37 posts (66%), with liver transplants being the most common (46%). Age at transplant was reported in 34 posts (61%), with 38% receiving their transplant at ages 0–9, 38% at 10–19, and 24% at 20+. Among those who reported years post‐transplant, 47% were in their first four years post‐transplant, while 53% had been living with their transplant for five or more years.

### 3.3 Major Post Categories and Sentiment

From all posts in our sample (*N* = 357), 189 (53%) were assigned to one of five major post categories, derived from the most frequently referenced personal experience codes from both SOT recipients and their family members. The remaining posts (47%) were often less distinct in topic (often addressing multiple areas related to medical topics, interpersonal relationships, and mental health challenges and their overlap) or did not fit neatly into the main thematic categories. These posts were not assigned to a specific theme, as they did not clearly delve into the emotional, social, or medical challenges that became the focus of our analysis. Relevant personal experiences captured within each category are depicted in Figure 2.

**FIGURE 2:**
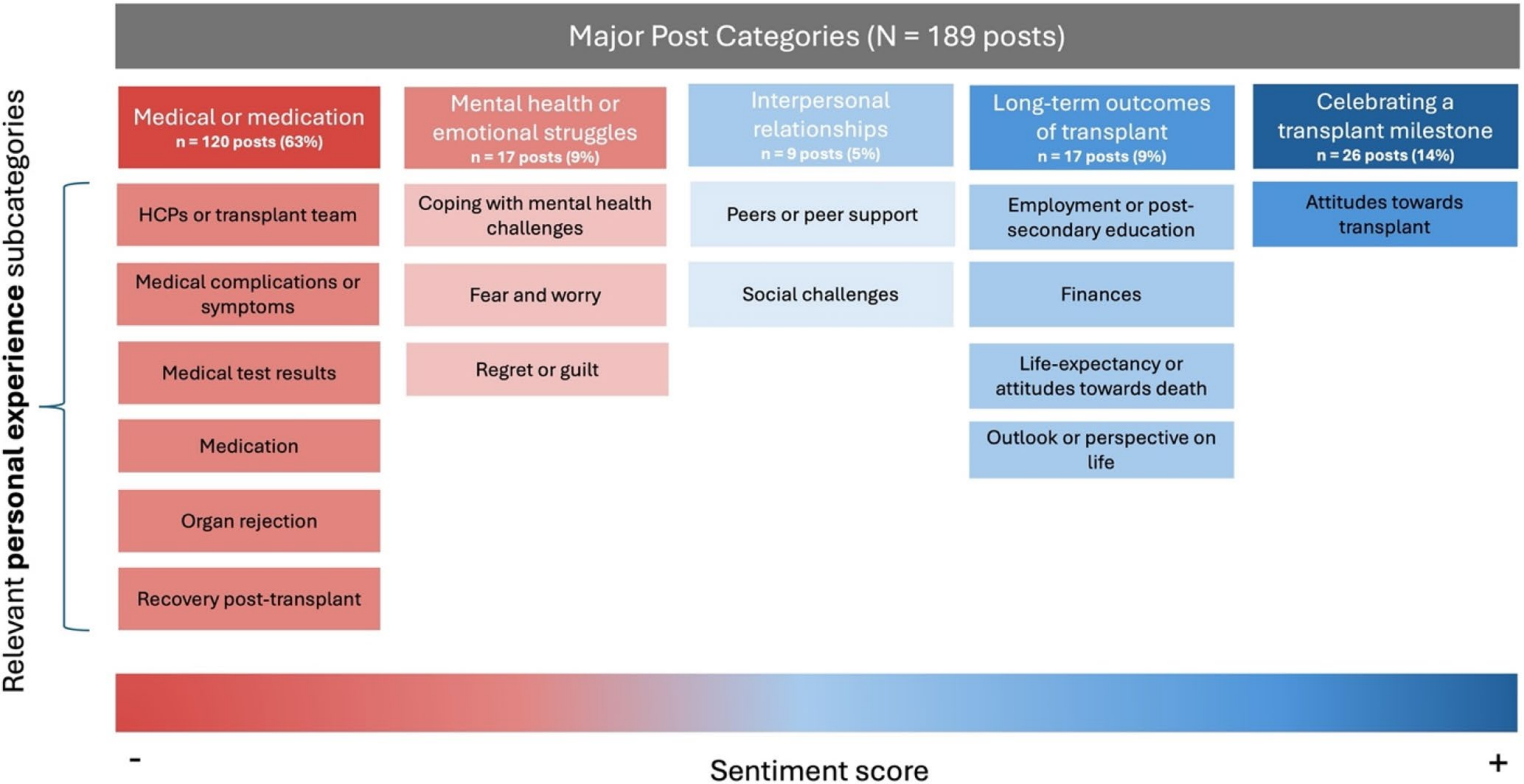
Five major post categories graded by mean sentiment score of post content. Relevant and frequently referenced personal experience subcategories were used to inform each major category. Scores range from more negative (represented in red) to more positive scores (represented in blue). The color intensity represents the mean sentiment score for each thematic cluster. Blue indicates a more positive average sentiment, while red indicates a more negative average sentiment. This is not a prescriptive categorization of each theme, but rather a data‐driven illustration of the average emotional valence associated with the language used in posts within each theme.

Of the 189 posts, the *Medical or medication* category made up 63% of posts (*n* = 120), *Mental health or emotional struggles* made up 9% of posts (*n* = 17), *Interpersonal relationships* made up 5% of posts (*n* = 9), *Long‐term outcomes of transplant* made up 9% of posts (*n* = 17), and *Celebrating a transplant milestone* made up 14% of posts (*n* = 26). There were 120 personal experience subcategories identified through the codebook development process. These generally branch off the major five post categories and are itemized in Figure 2. The following quotes illustrate content from posts in each major category. All quotes have been reviewed to ensure they do not contain any exact ages, unique dates, or other highly specific details that could be combined to identify a user. Any identifying words or phrases have been replaced by a broader category in square brackets.

1. *Medical or medication*

I will be getting my second kidney transplant in December. I had my first one when I was [age at transplant] years old and it lasted [duration of first transplant] years which is pretty good, I guess. Anyways, I wasn't really in charge of my life and getting ready for surgery at that time. Now I am. Any suggestions going into surgery to make the recovery go as smoothly as possible?

(Post 248)

2 *Mental health or emotional struggles*

Ever since I got my kidney transplant, I have felt guilty. Like I don't do enough or am not worthy of this second chance at life. I have been struggling with mental health for the past 2 years. It feels like everything I went through absolutely destroyed me…Does anyone else feel depressed?

(Post 150)

3 *Interpersonal relationships*

My friends had told my sister it was “too hard to be friends with someone who had a heart transplant” and apparently a lot of kids were jealous of the “special treatment” I was receiving and lots of people thought I had been faking all of my heart stuff for attention.

(Post 270)

4 *Long‐term outcomes of transplant*

Sometimes the kid(ney) and I have our dark days. We get depressed and wonder what the future holds for us. That's okay…We're not afraid about whatever uncertainties lie ahead of us, we're certainly not going to let our disability define us or what we do.

(Post 54)

5 *Celebrating a transplant milestone*.

A few days late, but I've now passed the [time since transplant]‐year mark with my new heart. I was about [age at transplant] old when I received the new heart. Hoping for another [time since transplant] years!

(Post 190)

To examine differences in the emotional tone of post content, sentiment scores were retrieved and are depicted for each category and subcategory in Figure 2. For the 189 posts that achieved categorization agreement, the mean sentiment score was −0.3 ± 11.1 and ranged from −75 to 37. Sentimentality differed significantly between categories (*p* = 0.015) (Figure 3). Posts categorized under *Celebrating a transplant milestone* were most positive in sentiment (*M* = 6.0 ± 8.3), posts about *Interpersonal relationships* showed the most neutral sentiment (*M* = 0.7 ± 7.7), and posts about *Long‐term outcomes of transplant* showed moderately positive sentiment (*M* = 2.1 ± 10.1). Posts about *Medical or medication* and *Mental health or emotional challenges* related topics were most negative (*M* = −1.9 ± 11.0 and *M* = −1.5 ± 14.5 respectively). Post hoc comparisons showed significantly more positive sentimentality for *Celebrating a transplant milestone* compared with *Medical or medication* (*p* < 0.01).

**FIGURE 3:**
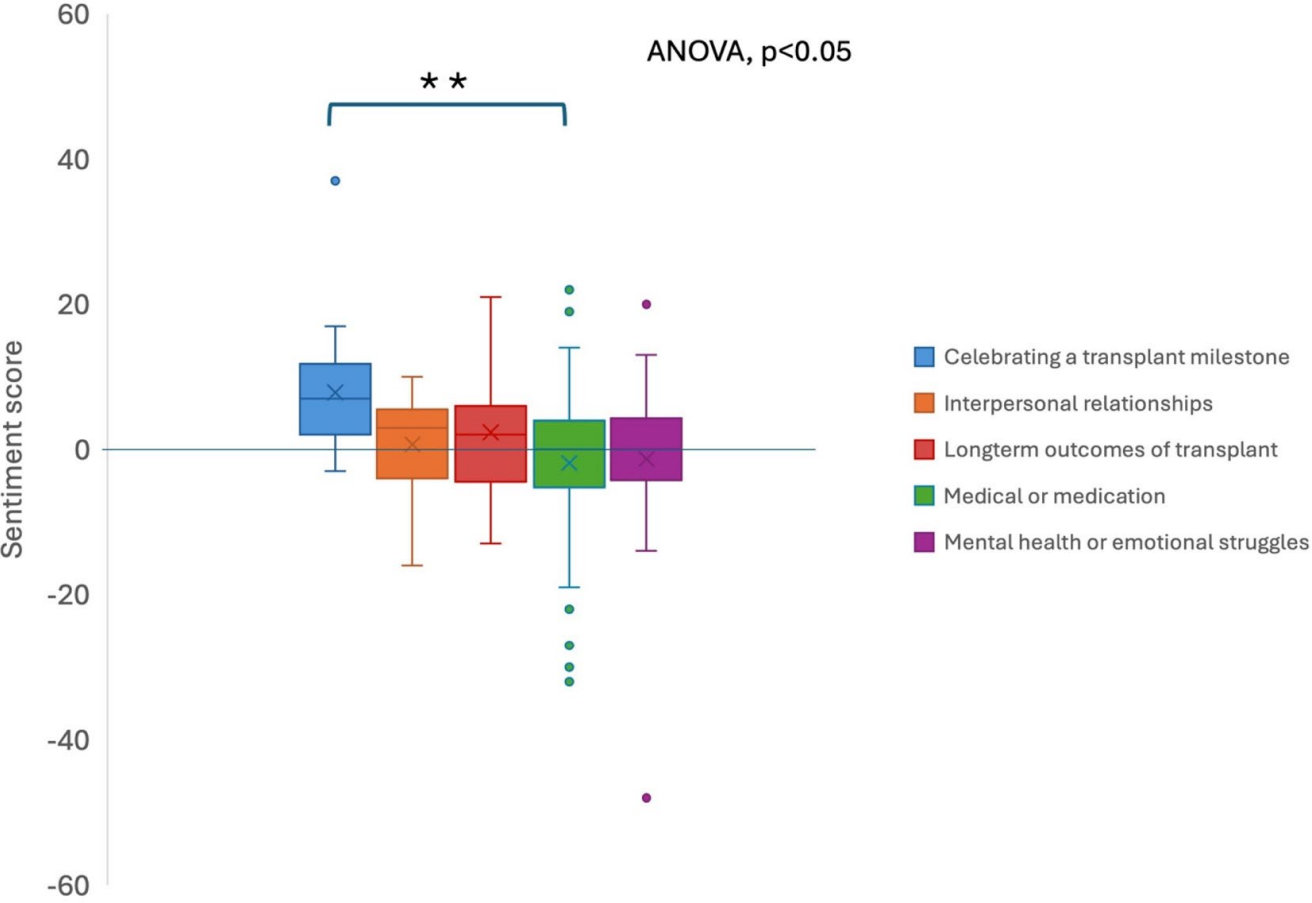
Sentiment score of major post categories: Celebrating a transplant milestone (*n* = 26 posts), Interpersonal relationships (*n* = 9 posts), Longterm outcomes of transplant (*n* = 17 posts), Medical or medication (*n* = 120 posts), and Mental health or emotional struggles (*n* = 17 posts). An ANOVA test followed by a Tukey's HSD was performed to assess for differences in sentiment score between categories. Significant differences are denoted by **p < 0.01.

#### 3.3.1 Information Seeking and Sharing

The most frequently sought topics by online users (posters and commenters) included medical complications or symptoms of transplant, medication, peers or social support, and HCPs or the transplant team (Figure 4). Topics shared most frequently addressed medical complications or symptoms and interactions with HCPs or the transplant team and also included themes related to mental health and safety.

**FIGURE 4:**
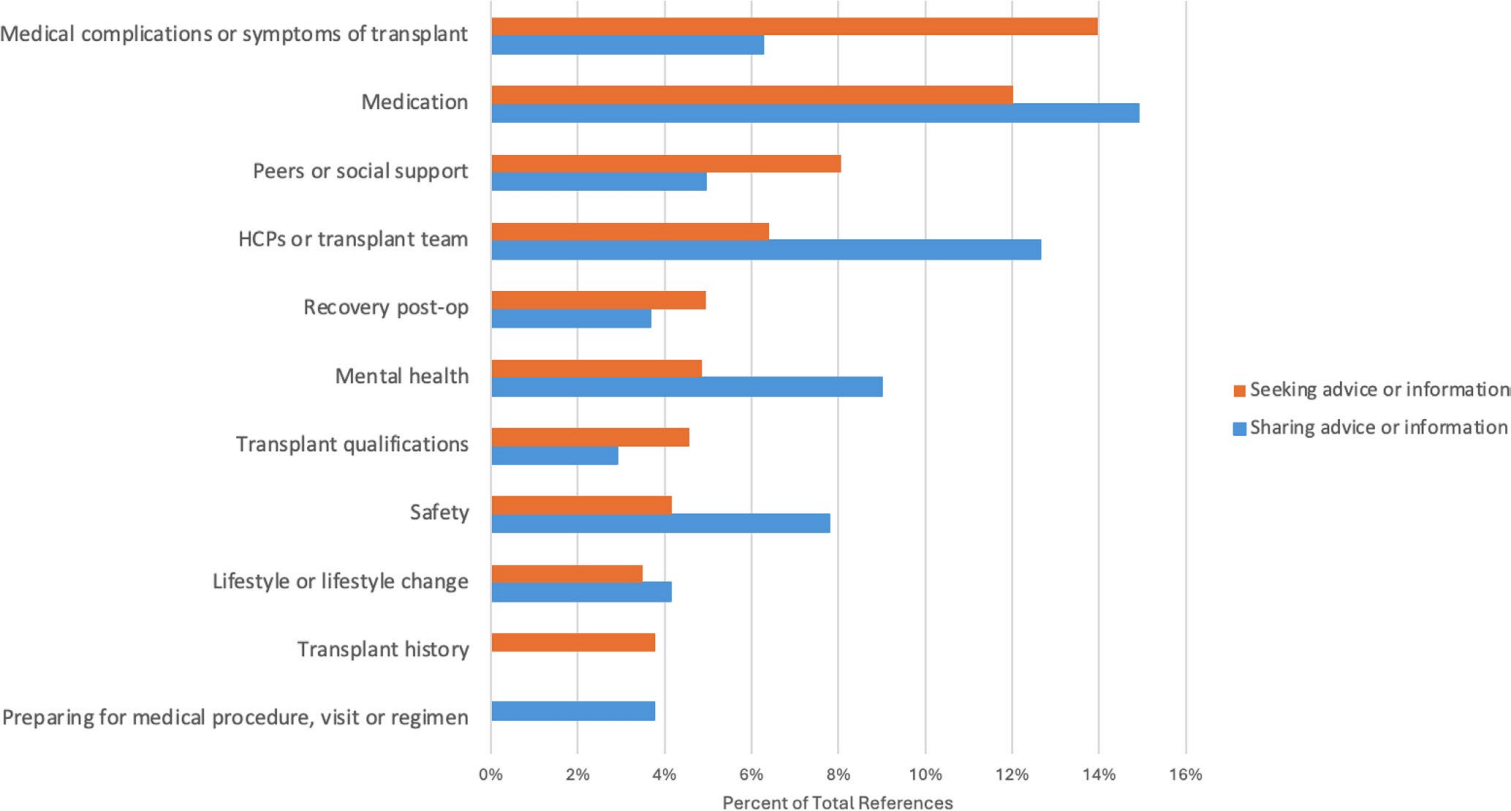
Most common topics for information or advice seeking or sharing by online users (posters and commenters). *N* = 1031 total references were included in the Seeking advice or information category and *N* = 3722 total references were included in the Sharing advice or information category. Percentages represent the proportion of references for each topic, calculated as: (number of references to a given topic/total references) × 100%.

### 3.4 Engagement With Posts in Major Categories

The mean number of comments varied significantly across categories (*p* < 0.05, Figure 5a), with *Long‐term outcomes of transplant* posts receiving the highest mean number of comments (*M* = 27.5 ± 22.7), followed by *Celebrating a transplant milestone* (*M* = 17.9 ± 9.2). Posts about *Medical or medication* related topics (*M* = 14.2 ± 12.8), *Interpersonal relationships* (*M* = 14.0 ± 14.6), and *Mental health or emotional struggles* (*M* = 13.6 ± 10.1) had the lowest mean number of comments. Post hoc comparisons showed that *Long‐term outcomes of transplant* posts received significantly more comments than *Medical or medication* (*p* < 0.01) and *Mental health or emotional struggles* (*p* = 0.025).

**FIGURE 5:**
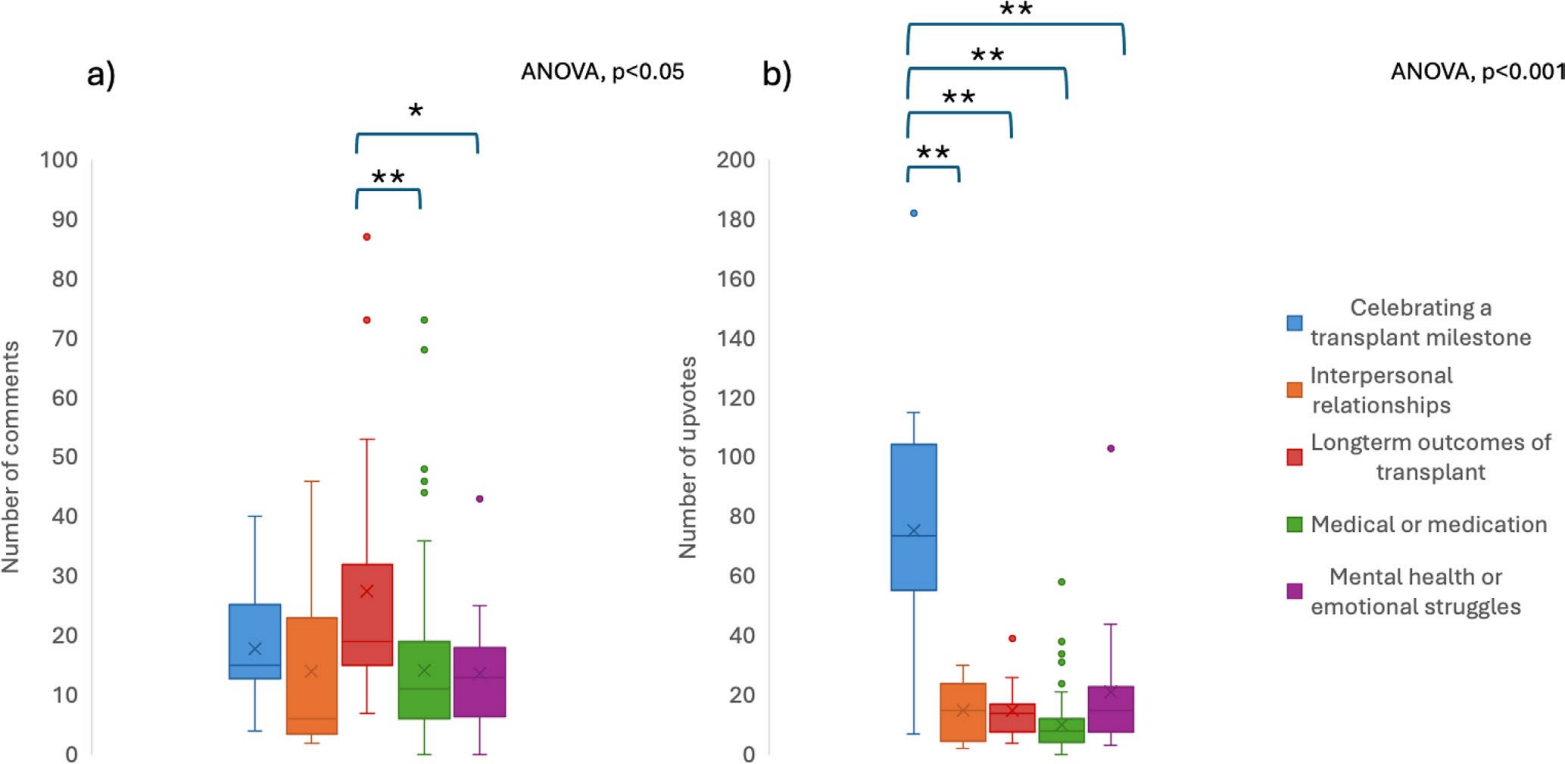
Number of (a) Comments and (b) Upvotes for major post categories on r/transplant. The five categories include: Celebrating a transplant milestone (*n* = 26 posts), Interpersonal relationships (*n* = 9 posts), Longterm outcomes of transplant (*n* = 17 posts), Medical or medication (*n* = 120 posts), and Mental health or emotional struggles (*n* = 17 posts). An ANOVA test followed by a Tukey's HSD was performed to assess for differences in comments and upvotes between categories. Significant differences are denoted by *p < 0.05 and **p < 0.01.

Number of upvotes also differed significantly depending on post category (*p* < 0.01, Figure 5b). *Celebrating a transplant milestone* posts received over three times the mean number of upvotes (*M* = 75.3 ± 36.8) than any other category: *Mental health or emotional struggles* (*M* = 20.9 ± 23.6), *Interpersonal relationships* (*M* = 14.9 ± 10.0), *Long‐term outcomes of transplant* (*M* = 14.9 ± 8.6), and *Medical or medication* (*M* = 10.0 ± 8.7) (*p* < 0.01 for all comparisons).

#### 3.4.1 Engagement With Posts Based on Demographic Characteristics

Differences in comments and upvotes were evaluated based on self‐disclosed demographic characteristics of users. Transplant recipients' posts received more comments than posts from family members (17.0 ± 12.9 vs. 12.7 ± 12.7, *p* = 0.02), whereas upvotes did not differ significantly between the two groups (20.7 ± 25.2 vs. 14.9 ± 21.4, *p* = 0.11) (Table 3). Among recipients, the number of comments did not vary by time since transplant (18.0 ± 11.6 for 0–4 years vs. 15.0 ± 10.3 for 5+ years, *p* = 0.12), but upvotes were higher for those 5+ years post‐transplant (27.7 ± 31.2 vs. 14.1 ± 13.0, *p* = 0.007). No significant differences in comments or upvotes emerged by gender, organ type, or age at transplant.

**TABLE 3:** Engagement with posts based on self‐disclosed demographic characteristics.

	Comments (mean, SD)	Upvotes (mean, SD)
Transplant recipient or family member		
Transplant recipient (*n* = 301)	17.0 (12.9)*	20.7 (25.2)
Family member (*n* = 56)	12.7 (12.7)	14.9 (21.4)
Gender		
Man or boy (*n* = 30)	18.1 (17.0)	13.7 (14.1)
Woman or girl (*n* = 28)	16.5 (8.7)	13.2 (11.8)
Organ type		
Kidney (*n* = 86)	15.7 (9.7)	21.1 (29.1)
Heart (*n* = 57)	15.5 (11.7)	21.0 (23.1)
Liver (*n* = 42)	19.1 (13.0)	22.7 (25.0)
Lung (*n* = 17)	18.6 (17.8)	22.4 (19.5)
Years post‐transplant		
0–4 years (*n* = 43)	18.0 (11.6)	14.1 (13.0)
5+ years (*n* = 105)	15.0 (10.3)	27.7 (31.2)*
Age at transplant		
0–9 years (*n* = 39)	17.8 (13.3)	31.5 (32.0)
10–19 years (*n* = 67)	20.1 (15.7)	28.2 (34.3)
20–24 years (*n* = 18)	18.2 (10.1)	20.4 (26.2)

* Statistically significant differences between groups (*p* < 0.05) was determined by an independent samples *t*‐test.

### 3.5 Categorization of Posts Based on User Characteristics

Difference in post frequency from SOT recipients (*n* = 301) and from self‐identified family members (*n* = 56) is summarized in Table 4. Post category distribution varied significantly between groups (*p* < 0.001, Table 4). Among transplant recipients, posts more commonly focused on *Medical or medication* (24%), *Mental health or emotional struggles* (21%), and *Celebrating a transplant milestone* (24%). In contrast, family members predominantly posted about *Medical or medication* (82%; *p* < 0.05), while none discussed *Mental health or emotional struggles* (*p* < 0.05). Posts about *Celebrating a transplant milestone* were also significantly less common among family members (4%) compared to recipients (24%) (*p* < 0.05).

**TABLE 4:** Categorization of posts from self‐disclosed transplant recipients (*n* = 301) and family members (*n* = 56).

Transplant recipient or family member	Transplant recipient	Family member
Post category^1^		
Included (matched)	147	28
Medical or medication	36 (24%)^a^	23 (82%)^b^
Mental health or emotional struggles	31 (21%)^a^	0 (0%)^b^
Interpersonal relationships	19 (13%)	3 (11%)
Long‐term outcomes of transplant	25 (17%)	1 (4%)
Celebrating a transplant milestone	36 (24%)^a^	1 (4%)^b^
Excluded (not matched or not in major category)	154	28

^1^ Distribution of posts across five major content categories was matched through coder agreement. A likelihood ratio chi‐square test (*χ* ^2^(4) = 39.8, *p* < 0.001) assessed differences in post category distributions. Superscripts (a, b) denote statistically significant differences between groups for individual categories (*z*‐test for proportions, *p* < 0.05).

Posts from SOT recipients were also stratified to represent those who were 0–4 years post‐transplant (*n* = 43) and 5+ post‐transplant (*n* = 105) (Table 5). Post categorization differed significantly (*p* < 0.001). *Medical or medication* was more common in the 0–4 year group (71%) than in the 5+ year group (51%), though not significantly. *Mental health or emotional struggles* were significantly more frequent in the 0–4 year group (25%) than the 5+ year group (7%, *p* < 0.05). In contrast, *Celebrating a transplant milestone* was significantly more common in the 5+ year group (32%) than the 0–4 year group (0%, *p* < 0.05).

**TABLE 5:** Categorization of posts from recipients that self‐disclosed as being 0–4 years (*n* = 43) or 5+ years (*n* = 105) post‐transplant.

Years post‐transplant	0–4 years	5+ years
Post category^1^		
Included (matched)	24	59
Medical or medication	17 (71%)	30 (51%)
Mental health or emotional struggles	6 (25%)^a^	4 (7%)^b^
Interpersonal relationships	0 (0%)	1 (2%)
Long‐term outcomes of transplant	1 (4%)	5 (8%)
Celebrating a transplant milestone	0 (0%)^a^	19 (32%)^b^
Excluded (not matched or not in major category)	19	46

^1^ Distribution of posts across five major content categories was matched through coder agreement. A likelihood ratio chi‐square test (*χ* ^2^(4) = 19.5, *p* < 0.001) assessed differences in post category distributions. Superscripts (a, b) denote statistically significant differences between groups for individual categories (*z*‐test for proportions, *p* < 0.05).

No significant differences in frequency of posts in major categories were observed between posts organized based on self‐disclosed gender (Man or boy; Woman or girl), type of organ received (Kidney; Heart; Liver; Lung), and age at transplant (0–9 years; 10–19 years; 20–24 years) (Tables S1–S3).

## 4 Discussion

The study aimed to understand how pediatric SOT recipients and their families engage with social media to share information and experiences. Users of the Reddit community r/transplant frequently sought and shared information about medical complications, medication, and psychosocial challenges associated with transplantation.

### 4.1 Pediatric SOT Recipients and Their Families Share Varying Categories of Posts Online, With Differing Emotional Tone and Engagement

Categorization and assessment of posts revealed distinct patterns: posts about medical complications or medication tended to have the most negative sentiment and received the lowest number of upvotes. This is further elaborated by a study from Zelikovsky and Nelson, which demonstrated in adolescent kidney transplant recipients that negative attitudes or perceptions about one's medication regimens contribute significantly to QoL [32]. These perceptions typically surrounded the possible overuse of medications or the negative impacts of their use. Research in adult dialysis patients that later received a transplant demonstrated that the substantial majority of patients believed their medications were necessary, but a proportion (34.4%) reported strong concerns [33].

In contrast, posts celebrating transplant milestones received the highest number of upvotes and were most positive in sentiment, suggesting that r/transplant engages with hopeful and affirming narratives. Posts sharing long‐term transplant outcomes, often about QoL, identity, and goals post‐transplant, had the highest number of comments. This may indicate that more discourse surrounds the broader and more existential aspects of post‐transplant life. These findings largely go against public perceptions of social media use in adolescents, including concerns about cyberbullying and hesitancy about sharing personal information due to privacy and security reasons [34, 35]. However, they do align with research suggesting that socializing and community building were common motivators for using Reddit [36]. Moreover, studies have demonstrated that young people with chronic disease use social media for peer support purposes, with participants sharing disease‐specific experiences on social media and receiving positive engagement [13, 37].

### 4.2 Patterns of Online Engagement Differ by User Role and Time Since Transplant

Family members were more likely to post about medical or medication‐related challenges, whereas recipients more often posted about mental health or emotional struggles, as well as milestone celebrations. This suggests that family members have unique informational needs, which may also be tied to a desire for interaction and support from others, similar to findings in parents of children with CKD [38]. The finding that family members, primarily parents, were more likely to post about health challenges may tie to previous research showing that poorer reports of their child's health were associated with greater PTS symptom severity in parents of transplant recipients [39]. Family functioning is also associated with trauma scores in pediatric SOT recipients, and families often report elevated stress and mental health symptoms post‐transplant, highlighting the need to address their unique support needs [40, 41].

Time since transplant was found to be a determinant in the type of post shared. Online users who were 0–4 years post‐transplant were more likely to post about mental health or emotional challenges, whereas those 5 or more years post‐transplant were more likely to post celebratory content. This aligns with existing literature suggesting that longer time since transplant is positively correlated with QoL, and that mental health domains of QoL often return to levels comparable to the general population over time [42, 43, 44]. This finding extends on previous studies that call for psychosocial interventions that address the interconnected needs of family members and recipients as they move through periods of adjustment post‐transplant [40, 45, 46].

### 4.3 Information Seeking and Sharing on r/Transplant Center Medical and Psychosocial Topics

Pediatric transplant recipients and their families turn to r/transplant to exchange information and navigate various post‐transplant challenges. The topics most frequently sought and shared online included medical complications, medication concerns, peer or social relationships, interactions with HCPs, recovery post‐transplant, and mental health. This online activity reflects prior research showing that transplant recipients and their caregivers often desire more information than they receive and that online sources are commonly consulted for their accessibility or when patients are hesitant to burden their healthcare providers [47, 48, 49, 50]. Specifically, concerns about complications and medication side effects have consistently emerged as principal information needs in this population, which is corroborated by the frequency that this topic was discussed on r/transplant [47]. Online discussions about HCPs are also well‐documented in other chronic disease populations, including tips for navigating the healthcare system [51, 52, 53]. In this context, information‐seeking is often a strategy for managing medical uncertainty, and supporting patients' understanding through education should be a priority [54].

When considering how HCPs might disseminate resources through social media, it is crucial to do this in a way that respects the patient‐led nature of these communities. Direct provider involvement in a space like r/transplant could fundamentally alter the dynamics, potentially making users hesitant to share openly. A more effective approach might involve sharing additional and related resources, such as providing links to materials in response to specific questions or creating separate platforms. In clinical settings, providers should focus on validating patients' desire to find community and support beyond local care environments.

### 4.4 Limitations and Future Directions

We acknowledge some limitations in the representation of the sample. The use of a single subreddit may not be representative of all online users in this population. Moreover, our findings represent the perspectives of a subset of active users on r/transplant that are willing to post. This does not capture the experiences of individuals who may browse content without posting, those who considered posting but decided against it, or those who do not use social media at all. These groups may have different needs and perspectives and should be the focus of future research. Our sample, therefore, represents active users who were willing to share their experiences publicly, which is a limitation inherent to observational social media research.

Our current analysis also focused on original posts rather than the comment threads, where users often shared medical advice based on personal experience or information passed on from their own medical teams. While users sometimes posted sharing medical advice, formally assessing the accuracy or quality of this advice was not within the scope of this project. Despite these limitations, findings from this study meaningfully contribute to our understanding of how this population engages on social media and underscores that social media can be a source of connectedness.

Future research should explore how to develop resources that directly address the gaps identified in our analysis. These include: (1) The need for accessible peer‐support networks that support connectedness and community among young transplant recipients; (2) The development of resources to address psychosocial challenges in young people living with a transplant, particularly in the post‐transplant period; and (3) The development of supports for family members and caregivers, particularly surrounding common medical complications or symptoms.

Moreover, while we did not contact users for this study to preserve the patient‐led nature of the space, we are exploring ethical ways to disseminate our findings back to the community. We plan to share the published paper on r/transplant and openly invite feedback and discussion from community members.

To our knowledge, this is the first study to evaluate patterns of social media activity in young people living with a SOT and their families. Findings provide critical insights into the topics this community engages with and the ways they interact with them, offering guidance for the development of targeted online informational and support tools.

## 5 Conclusion

The current study demonstrates how pediatric SOT recipients and their families engage with social media to seek information, share experiences, and connect with members of the community. Through a qualitative content analysis of posts and comments on a transplant‐specific online community, findings underscore that social media can be a valued source of self‐disclosure and information, specifically surrounding mental health, medical complications or medication, and long‐term aspects of the transplant journey. Differences in the content of posts shared by recipients versus family members, as well as by time since transplant, suggest different and evolving support needs across groups. Future research should explore the development of tailored online tools that address core informational and support needs of this population.

## Funding

This work was supported by Canadian Institutes of Health Research (178057).

## Conflicts of Interest

The authors declare no conflicts of interest.

## Supporting information


**Table S1:** Categorization of posts from transplant recipients self‐identifying as a man or boy (*n* = 30) or a woman or girl (*n* = 28).
**Table S2:** Categorization of posts from self‐disclosed kidney (*n* = 86), heart (*n* = 57), liver (*n* = 42), or lung (*n* = 17) recipients.
**Table S3:** Categorization of posts from recipients that self‐disclosed receiving a transplant between the years of 0–9 (*n* = 39), 10–19 (*n* = 67), or 20–24 (*n* = 18).
**Table S4:** Codes and subcodes under the categories of (1) Giving advice or information, (2) Seeking advice, information, stories, or experiences, (3) Personal experiences post‐transplant (user).

## Data Availability

The data that support the findings of this study are available in r/transplant at https://www.reddit.com/r/transplant/. These data were derived from the following resources available in the public domain: Reddit, https://www.reddit.com.
